# Impact of changes in commuting mode on body weight among Japanese workers: a longitudinal study

**DOI:** 10.1093/joccuh/uiae027

**Published:** 2024-05-24

**Authors:** Atsuko Fukunishi, Masaki Machida, Hiroyuki Kikuchi, Yutaka Nakanishi, Shigeru Inoue

**Affiliations:** Department of Preventive Medicine and Public Health, Tokyo Medical University, Tokyo, Japan; Department of Preventive Medicine and Public Health, Tokyo Medical University, Tokyo, Japan; Department of Preventive Medicine and Public Health, Tokyo Medical University, Tokyo, Japan; Daito Trust Construction Co., Ltd., Tokyo, Japan; Department of Preventive Medicine and Public Health, Tokyo Medical University, Tokyo, Japan

**Keywords:** active commuting, walking, public transport, worker

## Abstract

**Objective:**

The health benefits of active commuting have been reported. However, few studies have assessed commuting modes using objective methods. This study clarified the association between changes in objectively measured commuting modes and body weight among Japanese workers.

**Methods:**

This longitudinal study used data from the annual health examinations and personnel records of a company with branches in all prefectures of Japan. Data from 2018 and 2019 were used as the baseline and follow-up data, respectively. The commuting mode was assessed using the commuting mode code included in the personnel records and classified into 3 types: walking, public transport, and car or motorcycle. The participants were classified into 9 categories based on the combination of their commuting modes in 2018 and 2019. Body weight was measured objectively during health examinations. The 1-year changes in body weight were calculated for the 9 categories and assessed using an analysis of covariance with adjustments for covariates.

**Results:**

The analysis included 6551 workers (men: 86.8%; mean age: 42.8 years). Overall, body weights tended to increase (+0.40 kg/y). The participants who switched to more active commuting, such as from car or motorcycle to walking (−0.13 kg/y), from car or motorcycle to public transport (+0.10 kg/y), and from public transport to walking (−0.07 kg/y), exhibited small weight gains or losses. A similar trend was observed even after adjustment.

**Conclusions:**

Changing to a more active commuting mode may prevent weight gain among workers.

## Key points

- **What is already known on this topic:** The health benefits of active commuting or switching to active commuting have been reported. However, few studies have assessed commuting modes using objective methods.
- **What this study adds:** Objective changes to more active commuting, such as from car or motorcycle to walking, from car or motorcycle to public transport, and from public transport to walking, prevented weight gain among workers.
- **How this study might affect research, practice, or policy:** Commuting mode is related to workers’ health, especially body weight. This study suggests that considering the physical activity associated with commuting is important when managing workers’ health.

## Introduction

Physical activity is associated with various health benefits, including a lower risk of all-cause mortality, a reduced incidence of cardiovascular diseases and a few types of cancer, and improved mental health.^1–5^ Physical activity is performed in various domains, including leisure time, occupation, household, and/or transportation.^2^ Among workers, active commuting, namely travel to work by walking or cycling, has been considered as a way of increasing physical activity on weekdays,^6^^,^^7^ as suggested by the World Health Organization.^8^^,^^9^

Active commuting is associated with low all-cause mortality,^10^^-^^12^ and reduced risk of cardiovascular diseases,^10–13^ cancer,^10^ and diabetes.^10^^,^^14^ A recent systematic review showed that compared with inactive commuting, active commuting reduced the risk of obesity, hypertension, and diabetes by 12%, 5%, and 18%, respectively.^14^ However, many of the previous studies included in this systematic review were cross-sectional studies. Furthermore, only a small number of longitudinal studies focused on changes in commuting modes have been conducted recently.^15–18^ Previous longitudinal studies of the association between changes in commuting mode and body mass index (BMI) observed that switching from car commuting to commuting by walking, bicycling, or public transport was associated with low BMI, whereas a shift from commuting by walking, bicycling, or public transport to car commuting was associated with a high BMI.^15–17^ The follow-up period of these longitudinal studies ranged from approximately 2 to 5 years.^15–17^ However, for a changeable indicator such as body weight,^19^ the impact of changes in commuting mode may appear in a short period. Additionally, to the best of our knowledge, no study has assessed commuting modes using objective methods. Commuting patterns vary; however, there are no established methods for their evaluation. Most previous studies on the association between commuting mode and health outcomes, including these longitudinal studies, assessed commuting modes using original questionnaires or interviews that have not been validated.

Therefore, this 1-year longitudinal study aimed to clarify the association between changes in objectively measured commuting modes and body weight among Japanese workers.

## Methods

### Data collection and participants

This study used longitudinal data provided by a real estate company with branches in all prefectures of Japan. Data included annual health examinations conducted by the company and personnel records from the company in 2018 and 2019. We used health examination data conducted from May 2018 to December 2018 as baseline data and those performed from May 2019 to December 2019 as follow-up data. The health examination rates of this company in 2018 and 2019 were 100% excluding workers who were on leave or left the company, because the law requires workers to undergo health examinations at least once a year in Japan. The inclusion criterion was workers who underwent health examinations in 2018. Of these, participants with missing data on commuting mode, those with 2 or more types of commuting mode codes, and those with unclassifiable commuting mode codes in the 2018 dataset were excluded from the analysis. Additionally, participants who did not undergo health examinations in 2019, those with missing data on commuting mode, those with 2 or more types of commuting mode codes, and those with unclassifiable commuting mode codes in the 2019 dataset were also excluded.

### Ethics review

This study was approved by the Medical Ethics Committee of the Tokyo Medical University (approval no.: T2022-0005). This study was conducted with secondary use of existing data from a company. Therefore, consent for the use of the data was not obtained from the participants. Instead, a public announcement regarding opting out of this study was posted on the websites of the company and the Tokyo Medical University.

### Commuting mode

The commuting mode was assessed using the primary commuting mode code included in the personnel records. The company assigned each worker a primary commuting mode code, which was used to calculate the commuting allowance using a commuting management system. Commuting by a route that is not reasonable and shortest, or commuting using transportation that differs from the one applied for, may be regarded as an improper receipt of commuting allowances and subject to disciplinary action.^20^ The 13 types of primary commuting mode codes were classified into 3 modes: walking, public transport, and car or motorcycle (Table S1). The participants whose primary commuting mode codes were “private car or bicycle” were unclassified because we could not assess whether their modes were active or inactive.

### Body weight changes

During the annual health examinations in 2018 and 2019, body weight and height were measured using objective methods. We calculated body weight change (kg) by subtracting the body weight in 2018 from that in 2019.

### Covariates

We selected 7 covariates from the data provided by the company based on the previous studies on the associations between changes in commuting mode and BMI.^15–17^ Sex (men/women), age (years, continuous variable), position (staff/manager or above), and department (deskwork/nondeskwork) were obtained from the company personnel records in 2018. Deskwork included design and general affairs departments, which mainly included work in the office. Nondeskwork included sales and construction departments, which included work mainly out of the office. Data on smoking status (current smoker or non–current smoker), frequency of alcohol consumption (daily/sometimes or rarely), and perceived sleep status (adequate or inadequate rest) were obtained from a self-report questionnaire included in the 2018 health examination.

### Statistical analyses

The participants were categorized into 9 categories based on the combination of their commuting modes in 2018 and 1 year later. We considered the most active commuting mode to be walking, followed by public transportation and car or motorcycle based on previous studies.^21–23^ We further classified the 9 categories into 3 groups: (1) switching to active commuting, such as participants who switched their commuting modes from car or motorcycle to walking, from car or motorcycle to public transport, and from public transport to walking; (2) maintaining commuting mode in 2018; and (3) switching to inactive commuting, such as participants who switched from walking to public transport, from public transport to car or motorcycle, and from walking to car or motorcycle. The average body weight changes were calculated for all 9 categories. Additionally, body weight changes were assessed using analysis of covariance with adjustments for covariates (sex, age, position, department, smoking status, frequency of alcohol consumption, and perceived sleep status). All analyses were conducted using SPSS Statistics for Windows, version 29.0 software (IBM Japan, Tokyo, Japan).

## Results

**Figure 1:**
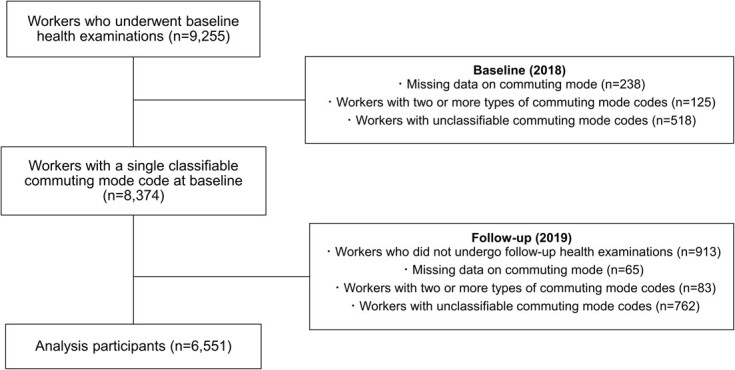
Participant flow.

Figure 1 shows the participant flow. A total of 9255 participants underwent health examinations in 2018. We excluded participants with missing data on commuting mode (*n* = 238), 2 or more types of commuting mode codes (*n* = 125), and unclassifiable commuting mode codes (*n* = 518) from the 2018 dataset. We further excluded participants who did not undergo health examinations in 2019 (*n* = 913), those who had missing data on commuting mode (*n* = 65), those with 2 or more types of commuting mode codes (*n* = 83), and those with unclassifiable commuting mode codes (*n* = 762) in the 2019 dataset. The final sample used for the analyses included 6551 participants.

Table 1 presents the participants’ characteristics. Among the 6551 participants, 5684 (86.8%) were men, with a mean age of 42.8 years. The baseline body weight was 70.6 kg, and the 1-year mean change in body weight was 0.4 kg. Regarding commuting modes, cars or motorcycles were the most common, followed by public transport and walking in both the baseline and follow-up surveys. Many participants (90.4%) maintained their baseline commuting mode. Those who switched to active commuting and inactive commuting accounted for 4.8% and 4.9%, respectively.

**Table 1:** Characteristics of participants.

**Characteristics**			**Participants (*n* = 6551)**			
			***n***	**%**	**Mean**	**SD**
**Sex**		**Men**	5684	86.8		
		**Women**	867	13.2		
**Baseline age**					42.8	10.6
**Position**		**Staff**	4838	73.9		
		**Manager or above**	1713	26.1		
**Department**	**Deskwork**	**Design**	1082	16.5		
		**General affairs**	1130	17.2		
	**Nondeskwork**	**Sales**	2790	42.6		
		**Construction**	1549	23.6		
**Smoking status**		**Current smoker**	2869	43.8		
		**Noncurrent smoker**	3682	56.2		
**Frequency of alcohol consumption**		**Daily**	2187	33.4		
		**Sometimes or rarely**	4364	66.6		
**Perceived sleep status**		**Adequate rest**	4031	61.5		
		**Inadequate rest**	2520	38.5		
**Baseline height, cm**					170.3	7.3
**Baseline body weight, kg**					70.6	13.2
**1-year change in body weight, kg**					0.4	2.9
**Commuting mode**	**At baseline**	**Walking**	670	10.2		
		**Public transport**	2368	36.1		
		**Car or motorcycle**	3513	53.6		
	**At follow-up**	**Walking**	614	9.4		
		**Public transport**	2451	37.4		
		**Car or motorcycle**	3486	53.2		
**Change in commuting mode**	**Switching to active commuting**	**From car or motorcycle to walking**	84	1.3		
		**From car or motorcycle to public transport**	153	2.3		
		**From public transport to walking**	75	1.1		
	**Maintained commuting mode**	**Walking**	455	6.9		
		**Public transport**	2190	33.4		
		**Car or motorcycle**	3276	50.0		
	**Switching to inactive commuting**	**From walking to public transport**	108	1.6		
		**From public transport to car or motorcycle**	103	1.6		
		**From walking to car or motorcycle**	107	1.6		

**Figure 2:**
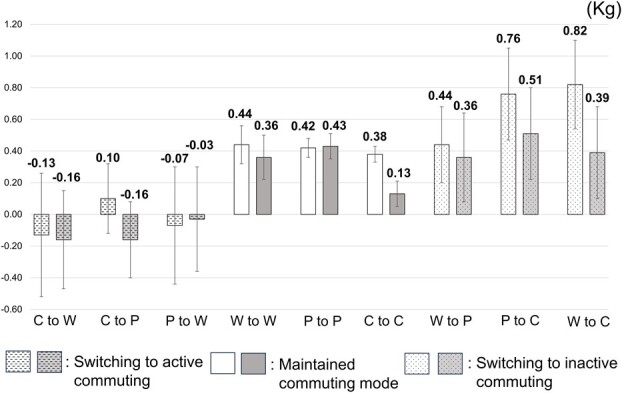
Mean body weight changes and estimated mean body weight changes for each commuting mode change. W: Walking; P: Public transport; C: Car or motorcycle. White bars represent the mean body weight changes. Gray bars represent the estimated mean body weight changes. The estimated mean body weight changes are adjusted for sex, age, position, department, smoking status, frequency of alcohol consumption, and perceived sleep status. Error bars represent standard error of the mean.

Figure 2 shows the mean body weight changes and the estimated mean body weight changes in the 9 categories. The maintained commuting mode group had a weight gain of approximately 0.4 kg (range: +0.38 to +0.44 kg) before adjustment. The participants who switched to more active commuting experienced a small weight gain or weight loss (range: −0.13 to +0.10 kg). Even after adjustment for covariates, this trend was observed (range: −0.16 to −0.03 kg).

## Discussion

In this study, we investigated the impact of changes in commuting modes on the body weight of Japanese workers using annual health examination data and objectively measured commuting data provided by a company. During the 1-year follow-up period, participants who switched to more active commuting experienced slight weight gains or weight losses. This trend persisted after adjusting for covariates.

Overall, the 1-year change in body weight was +0.40 kg on average. In the group that maintained commuting mode at baseline, identical weight gains were observed (range: +0.38 to +0.44 kg) regardless of the type of commuting mode. A study of Japanese workers reported that male workers who did not improve their lifestyle, including eating and exercise habits, gained a mean of 0.41 kg for 1 year, which was similar to the mean weight change observed in this study.^24^

A 4.4-year longitudinal study of British workers in mid-life showed that, compared with maintained car commuting, switching from car commuting to active or public transport commuting was associated with a lower BMI of 0.30 kg/m^2^.^16^ Moreover, a 2-year longitudinal study in the United Kingdom revealed similar findings.^15^ A longitudinal study of Japanese workers in a single company indicated that the change in BMI over 5 years was +0.19 kg/m^2^ for adults who maintained car commuting, whereas it was +0.10 kg/m^2^ for adults who switched from car commuting to active or public transport commuting.^17^ On converting body weight changes observed in this study to BMI (converted by the average height of the participants in this study [170.3 cm]), the changes in BMI were +0.13 kg/m^2^ (+0.38 kg) for participants who maintained car or motorcycle commuting. Meanwhile, it was −0.04 kg/m^2^ (−0.13 kg) for participants who switched from commuting by car or motorcycle to walking, and +0.03 kg/m^2^ (+0.10 kg) for participants who switched from commuting by car or motorcycle to public transport. Although the amount of weight changes differed due to the differences in the follow-up period or categories of commuting mode, our findings that the change to active commuting modes prevented weight gain were consistent with the results of previous studies.

The intensity of physical activity in different commuting modes can be expressed in metabolic equivalents (METs), which is a physiological measure; 1 MET is defined as the energy expenditure while sitting at rest, and an activity with a MET value of 3 requires 3 times the resting expenditure.^2^ According to the 2011 Compendium of Physical Activities, the METs by means of transportation are as follows: walking to work = 4.0 METs; automobile or light truck driving = 2.5 METs; and riding a bus or train = 1.3 METs.^25^ In this compendium, the intensity of riding a bus or train is lower than that of automobile driving. However, a previous study reported that train commuters engaged in walking from their home and workplaces to the station, making them more physically active during their commute than car commuters.^26^ Considering these data, changes to active commuting might have increased physical activity during commuting, resulting in weight gain prevention.

Additionally, our study results suggest that changes in commuting mode may have an impact on body weight, even within a short period, such as within 1 year. In the present study, many of the changes in commuting modes likely occurred in April 2019, when personnel changes occurred. However, 99.5% of the participants underwent follow-up health examinations by July 2019. Considering the timing of the health examination and personnel changes, many participants who switched their commuting mode would have used it for approximately 1 to 4 months. Changes in commuting mode may affect body weight even within a short period of a few months.

The strength of this study is that we used objectively measured commuting modes and body weights for the analysis. To the best of our knowledge, no study has measured commuting modes using objective methods. However, this study has several limitations. First, approximately 30% of participants were excluded from the analysis. Among those excluded, participants who did not undergo a follow-up examination (*n* = 913, 33.8%) were considered to have left the company in 2018, as the examination rates in 2018 and 2019 were 100%. Therefore, the results of this study may have been influenced by healthy worker bias. Second, we did not obtain data on the reasons for the changes in commuting modes. If the commuting mode changes due to job relocation, body weight may be significantly affected by lifestyle changes such as diet, exercise, and treatment status. Third, we could not quantitatively assess commuting because we did not have data on commuting distance or commuting time. Fourth, the possibility of residual confounders, such as mental indicators, that might affect the results, could not be excluded. Fifth, because the present study was conducted at a single company, its generalizability is limited. Despite these limitations, this study suggests that commuting mode plays an important role in the health management of workers, especially in body weight control.

In conclusion, this study revealed that switching to a more active commuting mode prevents weight gain among workers. Promoting physical activity is important for workers’ health. However, few guidelines or policies on occupational health have focused on commuting as a place to practice physical activity.^27^ It is important to consider the physical activity associated with commuting when managing workers’ health.

## Supplementary Material

Web_Material_uiae027

## Data Availability

The data cannot be shared publicly because of the confidentiality-based restrictions placed by the Ethics Committee at Tokyo Medical University. The datasets used and/or analyzed during the present study are available from the corresponding author upon reasonable request.
