# Supplementary material for: Impact of changes in commuting mode on body weight among Japanese workers: a longitudinal study
Source: J Occup Health. 2024 May 24;66(1):uiae027. doi: 10.1093/joccuh/uiae027 (PMC11285781; doi:10.1093/joccuh/uiae027)
Supplement: Web_Material_uiae027 [file web_material_uiae027.pdf]

Supplement Table 1. Classification of primary commuting mode codes

Primary commuting mode code (English)	Primary commuting mode code (Japanese)	Commuting mode
Walking	徒歩	Walking
Transportation-monthly basis*	交通機関-月額	Public Transport
Transportation-daily basis†	交通機関-日額	Public Transport
Transportation-monthly basis (manual input‡)	交通機関-月額（手入力）	Public Transport
Transportation-daily basis (manual input)	交通機関-日額（手入力）	Public Transport
Bus-monthly basis	バス-月額	Public Transport
Bus-monthly basis (manual input)	バス-月額（手入力）	Public Transport
Fixed allowance§ (Public transport)	固定額（公共交通機関)	Public Transport
Fixed allowance (Public transport)-daily basis	固定額（公共交通機関)-日額	Public Transport
Company car	社用車	Car or motorcycle
Fixed allowance (Private car)	固定額（私有車）	Car or motorcycle
Private car or bicycle-monthly basis	私有車・自転車-月額	Unclassified
Private car or bicycle-daily basis	私有車・自転車-日額	Unclassified

Commuting mode codes include terms related to the commuting mode, calculation of commuting allowances, and input of commuting data.

Walking, transportation, bus, public transport, company car, private car, and bicycle in the code names are terms denoting commuting modes.

*†§ Monthly basis, daily basis, and fixed allowance are terms related to the calculation of commuting allowances.

‡ Manual input is a term related to the input of commuting data.
